# Supplementary material for: Natural Killer Cell Receptors and Endometriosis: A Systematic Review
Source: Int J Mol Sci. 2022 Dec 25;24(1):331. doi: 10.3390/ijms24010331 (PMC9820702; doi:10.3390/ijms24010331)
Supplement: Supplementary file 1 [file ijms-24-00331-s001.zip › Table S1 - Search Strategy.pdf]

Table S1. Search strategy

Query	Search
**PubMed/Medline**	
#1	(endometriosis[MeSH Terms]) OR (Endometrioses) OR (Endometrioma) OR (Endometriotic) OR (ectopic endometrium)
#2	(cell, natural killer[MeSH Terms]) OR (NK cell) OR (Natural killer cell) OR (natural killer cell receptor)
#3	#1 AND #2
**EMBASE**	
#1	('endometriosis'/exp OR 'ectopic endometrium'/exp OR 'endometriosis' OR 'endometrioma' OR 'endometriotic' OR 'ectopic endometrium' OR 'endometrioses' OR 'endometriosis externa')
#2	('natural killer cell'/exp OR 'natural killer cell' OR 'nk cell' OR 'natural killer cell receptor')
#3	#1 AND #2
**Scopus**	
#1	TITLE-ABS-KEY ("endometriosis") OR TITLE-ABS-KEY("Endometrioses") OR TITLE-ABS- KEY("Endometrioma") OR TITLE-ABS-KEY("Endometriotic") OR TITLE-ABS-KEY("ectopic endometrium")
#2	TITLE-ABS-KEY("NK cell") OR TITLE-ABS-KEY("Natural killer cell") OR TITLE-ABS-KEY("natural killer cell receptor")
#3	#1 AND #2
**Web of Science**	
#1	TS=("endometriosis" OR "Endometrioses" OR "Endometrioma" OR "Endometriotic" OR "ectopic endometrium")
#2	TS=("NK cell" OR "Natural killer cell" OR "natural killer cell receptor")
#3	#1 AND #2

MeSH, Medical Subject Headings.
