# Supplementary material for: Natural Killer Cell Receptors and Endometriosis: A Systematic Review
Source: Int J Mol Sci. 2022 Dec 25;24(1):331. doi: 10.3390/ijms24010331 (PMC9820702; doi:10.3390/ijms24010331)
Supplement: Supplementary file 1 [file ijms-24-00331-s001.zip › Table S2 - Eligibility criteria and study selection_updated.pdf]

Reference	Inclusion criteria	Exclusion criteria
Wu et al., 2000 [5]	Fertile women undergoing laparoscopy for endometriosis (experimental group) OR tubal ligation (control group)<br>Early follicular phase after menstruation	Hormonal treatment <3 months prior laparoscopic operation<br>Evidence of inflammation/endometriosis in control group
Hornung et al., 2001 [6]	Normally menstruating women undergoing laparoscopy for endometriosis (experimental group) OR infertility evaluation, pelvic pain, pelvic mass, or elective tubal sterilization (control group)<br>Proliferative phase of menstrual cycle	Oral contraceptives or GnRH analogues <3 months prior laparoscopic operation
Maeda, Izumiya, Oguri, et al., 2002 [7]	Women undergoing laparoscopy for endometriosis (experimental group) OR benign ovarian cysts, uterine myoma, infertility evaluation, paraovarian cysts, or carcinoma in situ of uterine cervix (control group)<br>Early follicular phase	History of pregnancy <3 years prior to laparoscopic operation<br>History of GnRH analogue treatment <3 years prior to laparoscopic operation<br>Suspicion of associated complications of pelvic inflammatory disease
Maeda, Izumiya, Yamamoto, et al., 2002 [8]	Women undergoing laparoscopy for endometriosis (experimental group) OR benign ovarian cysts, uterine myoma, infertility evaluation, paraovarian cysts, or carcinoma in situ of uterine cervix (control group)	History of pregnancy <3 years prior to laparoscopic operation<br>History of GnRH analogue treatment <3 years prior to laparoscopic operation<br>Suspicion of associated complications of pelvic inflammatory disease
Maeda et al., 2004 [9]	Japanese women undergoing laparoscopy for endometriosis (experimental group) OR uterine myoma, benign ovarian cysts, infertility evaluation, paraovarian cysts, or chronic abdominal pain (control group)<br>Follicular and Luteal phase	History of pregnancy <1 year prior to laparoscopic operation<br>History of GnRH analogue treatment <1 year prior to laparoscopic operation<br>Suspicion of associated complications of pelvic inflammatory disease
Matsuoka et al., 2005 [10]	Japanese women undergoing laparoscopy for endometriosis (experimental group) OR uterine myoma, benign ovarian cysts, infertility evaluation, paraovarian cysts, or chronic abdominal pain (control group)<br>Follicular and Luteal phase	History of pregnancy <1 year prior to laparoscopic operation<br>History of GnRH analogue treatment <1 year prior to laparoscopic operation<br>Suspicion of associated complications of pelvic inflammatory disease
Barrier et al., 2006 [12]	Peritoneal endometriotic lesions and eutopic endometrium samples with confirmed endometriosis (unknown patient clinical history)<br>Eutopic endometrial samples without endometriosis from proliferative and secretory phase (unknown patient clinical history)<br>Eutopic endometrial biopsies from women with endometriosis and women without endometriosis undergoing elective laparoscopic sterilization (in proliferative and secretory phase)<br>Women with regular menstrual cycles	History of reproductive steroid-modulating medications within the past 6 months in biopsy/laparoscopic group
Eidukaite et al., 2006 [11]	Women complaining of abdominal pain with and without endometriosis<br>Proliferative phase<br>24-39 years old	Evident changes in abdominal cavity (except endometriosis)<br>Unclear whether hormonal treatment was considered
Eidukaite and Tamosiunas, 2008 [13]	Women complaining of abdominal pain with and without endometriosis<br>Proliferative phase<br>24-39 years old	Unclear/Not reported
Galandrini et al., 2008 [14]	21-48 years old<br>Women undergoing laparoscopy for endometriosis (experimental group) OR ovarian endometrioma (control group)	Oral contraceptives or GnRH analogues <3 months prior laparoscopic operation<br>Endocrine, immunologic, neoplastic, or other chronic diseases<br>Suspicion of associated complications of pelvic inflammatory disease
Baka et al., 2011 [15]	Women of reproductive age with endometriosis undergoing curettage (experimental group) OR women (without endometriosis) undergoing hysterectomy due to persistent high grade cervical intraepithelial neoplasia<br>22-45 years old	Autoimmune diseases or previous immune therapy
Funamizu et al., 2014 [16]	Women undergoing laparoscopy for endometriosis (experimental group) OR benign ovarian cysts, uterine myoma (control group)<br>Proliferative, secretory and anovulatory phase	Mild (stage I and II) endometriosis
González-Foruria et al., 2015 [17]	< 42 years old<br>Women undergoing laparoscopy for endometriosis (experimental group) OR benign gynecological diseases (control group)	Pregnancy<br>Malignancy/cancer
Rached et al., 2019 [18]	Women with regular menstrual periods who underwent laparoscopy for endometriosis (experimental group) OR elective tubal ligation (control group)	Oral contraceptives or GnRH analogues <3 months prior laparoscopic operation<br>Existence of other inflammatory, autoimmune, or malignant disease<br>Histological inflammatory signs in eutopic endometrium samples
Wu et al., 2019 [24]	Women undergoing hysterectomy for endometriosis (experimental group) OR benign gynecological conditions (control group)<br>Regular menstrual cycles	Other conditions that affect the endometrium<br>Oral contraceptives or GnRH analogues <3 months prior laparoscopic operation
Xu, 2019 [19]	22-49 years old women undergoing laparoscopy or laparotomy due to endometriosis (experimental group) OR ovarian cysts (control group)<br>Regular menstrual cycles<br>Proliferative or secretory phase	Oral contraceptives or GnRH analogues <3 months prior laparoscopic operation<br>Immunological disorders
Chou et al., 2020 [20]	Han Chinese women undergoing laparoscopy due to endometriosis (experimental group) OR myoma, teratoma, serous cystadenoma, ovarian cyst, ovarian stroma, dermoid cyst, mucinous cystadenoma, paratubal cyst, follicular cyst, simple cyst, hydrosalpinx, corpus luteum cyst, fibrous adhesion, and struma ovarii (control group)	Autoimmune disorders
Saeki et al., 2022 [25]	Premenopausal women who have given informed consent, undergoing laparoscopy due to endometriosis (experimental group) OR non-endometrioid benign ovarian cysts, leiomyoma	Immunological disorders

Table S2. Eligibility criteria and study selection
